# Case report: A case of corneal epithelial injury associated with *Pthiriasis palpebrarum*

**DOI:** 10.3389/fmed.2022.955052

**Published:** 2022-08-01

**Authors:** Da-Hu Wang, Xin-Quan Liu

**Affiliations:** Department of Ophthalmology, Longhua Hospital Affiliated to Shanghai University of Traditional Chinese Medicine, Shanghai, China

**Keywords:** *Pthirus pubis*, *Pthiriasis palpebrarum*, corneal epithelial injury, blepharitis, dry eye disease, case report

## Abstract

**Significance:**

*Phthiriasis palpebrarum* is an uncommon infection due to *Phthirus pubis* inoculating the eyelashes and surrounding tissues of the eye. Because of its rarity, it may be misdiagnosed as blepharitis or conjunctivitis clinically.

**Purpose:**

This report described a rare case of corneal epithelial injury associated with *Phthiriasis palpebrarum*.

**Case report:**

A 59-year-old woman presented with 1 month history of repeated episodes of itching and irritation symptoms in both eyes. A slit-lamp examination was performed, which revealed mild conjunctival hyperemia and corneal epithelial defects in both eyes. On closer examination, crab-like lice, nits, and red pinpoint excretions were seen on her eyelashes and eyelids bilaterally. Corneal fluorescein staining in both eyes was observed, and tear film break-up time (BUT) in each eye was 2 s. Numerous lice were also found attached to the scalp hair. Therefore, a clinical diagnosis of corneal epithelial injury associated with *Pthiriasis palpebrarum* was made. For treatment, eyelashes with nits and/or lice were removed mechanically with a fine tweezers. Then, 0.01% Hypochlorous Acid eye wash was used to clean the eyelid margin twice daily. Also, she was prescribed a combination of Vitamin A Palmitate eye gel three times a day and Tobradex^®^ eye ointment once daily. Meanwhile, the patient was provided with suggestions on how to improve personal hygiene and environmental hygiene, including cutting of the scalp hair and the application of 0.01% permethrin rinse. One week later, no evidence of lice and nits of the eyelashes and scalp hair was found, and the patient's symptoms and signs also improved significantly.

**Conclusion:**

This rare case suggested that the eyelashes of patients presenting with itching and irritation symptoms should be carefully examined with a slit-lamp. Besides removal of the parasites, attention should be paid to the treatment of corneal epithelial injury associated with *Pthiriasis palpebrarum*.

## Introduction

*Pthirus pubis* (sometimes written *Phthirus pubis*) is commonly known as the pubic louse or crab louse (1, 2), which is morphologically different from *Pediculus humanus capitis* (head louse) and *Pediculus humanus corporis* (body louse) (2–4). It is generally spread through close contact, but also through contaminated towels, pillows, toilet seats, clothing or bedding (1–3). Although it usually infests the pubis, the involvement of the eyelashes, eyebrows and scalp has occasionally been observed (1, 2, 4–8).

*Pthiriasis* of the eyelashes, also named *Pthiriasis palpebrarum* (1, 4), is more common in children (6, 7, 9–11), but it can also be observed in adults (1–18). *Pthiriasis palpebrarum* usually occurs in both eyelids (1). Pruritus (itching) is the most frequent symptom (2, 4), and the most common clinical presentation is the finding of numerous nits stuck to the eyelashes (1, 2, 4).

If nits and pinpoint excretions were not observed on the eyelashes and eyelids at the initial examination, *Pthiriasis palpebrarum* is often difficult to identify. In addition, its symptoms and signs are similar to those of conjunctivitis, blepharoconjunctivitis or blepharitis, so it is easily overlooked (10–15).

At present, there are few reports of corneal lesions in infected persons, so we report a case of corneal epithelial injury associated with *Pthiriasis palpebrarum* in an adult woman.

## Case report

### Initial examination

**Figure 1:**
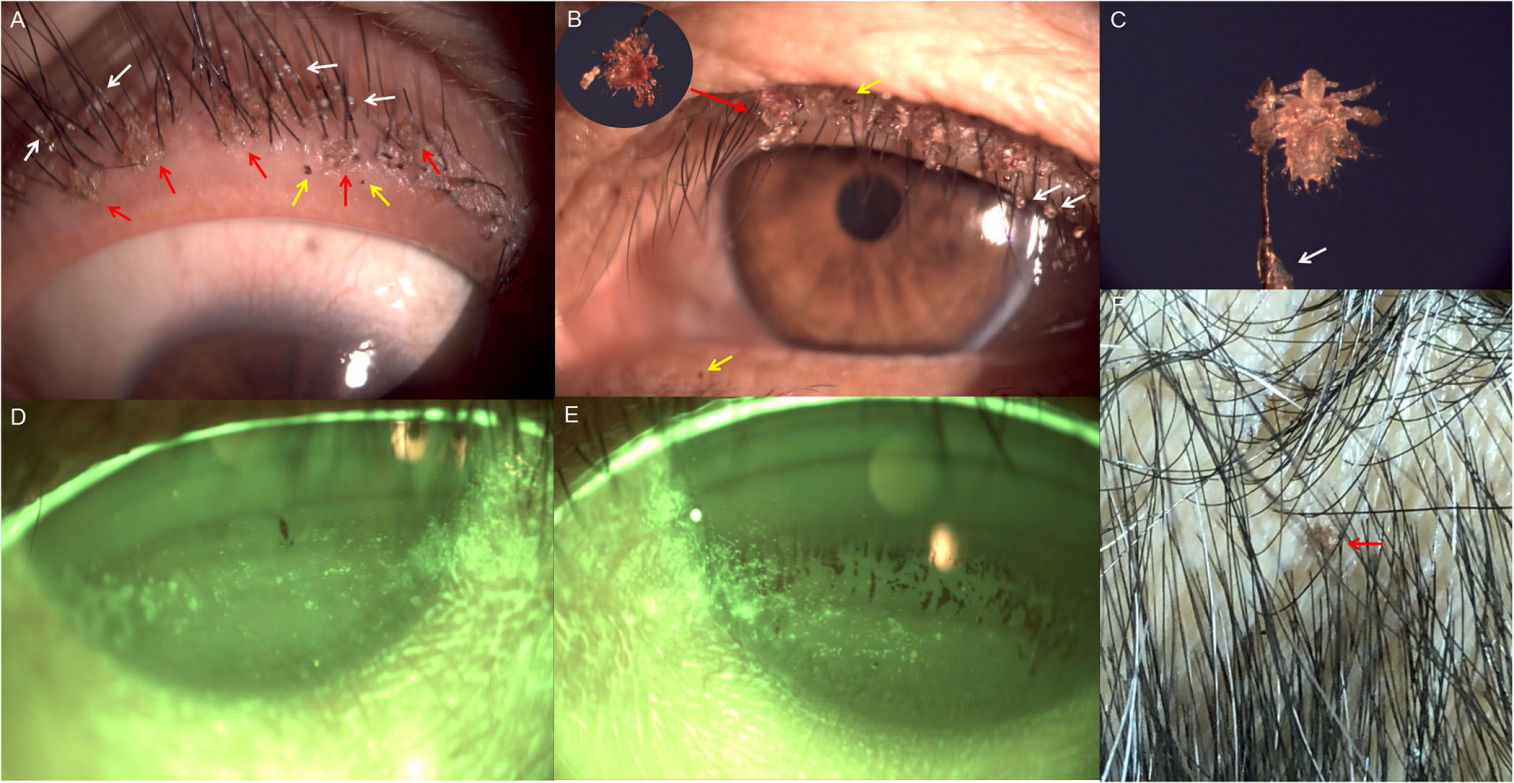
Slit-lamp photographs of both eyes and smartphone photograph of the scalp at the initial examination. **(A,B)** Lice (red arrows) and fecal matter (yellow arrows) on the lid margin of both eyes; numerous nits firmly stuck to the eyelashes (white arrows). **(C)** Crab-like louse grasping the eyelash (40 × magnification); translucent nit adhering to the eyelash (white arrow). **(D)** Corneal epithelial defects of right eye. **(E)** Corneal epithelial defects of left eye. **(F)** Louse (red arrow) on the scalp.

A 59-year-old woman visited the Department of Ophthalmology, Longhua Hospital Affiliated to Shanghai University of Traditional Chinese Medicine on Oct 21, 2021, presenting with 1 month history of repeated episodes of itching and irritation symptoms in both eyes. She said her 3-year-old granddaughter had similar symptoms and was diagnosed with *Pthirus pubis* infection of the eyelashes and scalp in other hospital a week ago. A slit-lamp examination was performed, which revealed mild conjunctival hyperemia and corneal epithelial defects in both eyes. On closer examination, crab-like lice, nits, and red pinpoint excretions (fecal matter) were seen on her eyelashes and eyelids bilaterally (Figure 1). Corneal fluorescein staining in both eyes was observed, and tear film break-up time (BUT) in each eye was 2 s. Numerous lice were also found attached to the scalp hair. At the same time, we found that her granddaughter's hair was shaved off, and no abnormality was found in her eyelids. Therefore, a clinical diagnosis of corneal epithelial injury associated with *Pthiriasis palpebrarum* was made. In addition, the patient stated that she lived with her granddaughter for 3 years, and had a history of taking care of her bedridden father for more than 2 years and had no history of sexually transmitted diseases. Then, she was referred to the dermatological department for further evaluation, and the pubic hair was found to be free from infestation.

For treatment, eyelashes with nits and/or lice were removed mechanically with a fine tweezers (see Video, Supplemental digital content 1, which demonstrates the removal of eyelashes with nits and/or lice). Then, 0.01% Hypochlorous Acid eye wash (2ml:0.2mg, Suzhou Yurou Medical Instrument Co., Ltd, Suzhou, China) was used to clean the eyelid margin twice daily. Also, she was prescribed a combination of Vitamin A Palmitate eye gel (Shenyang Xingqi Pharmaceutical Co., Ltd, Shenyang, China) three times a day and Tobradex^®^ eye ointment (tobramycin 0.3%/dexamethasone 0.1%, Alcon) once a day. Meanwhile, the patient was provided with suggestions on how to improve personal hygiene and environmental hygiene, including cutting of the scalp hair and the application of 0.01% permethrin rinse (pediculicide).

### Follow-up visits

**Figure 2:**
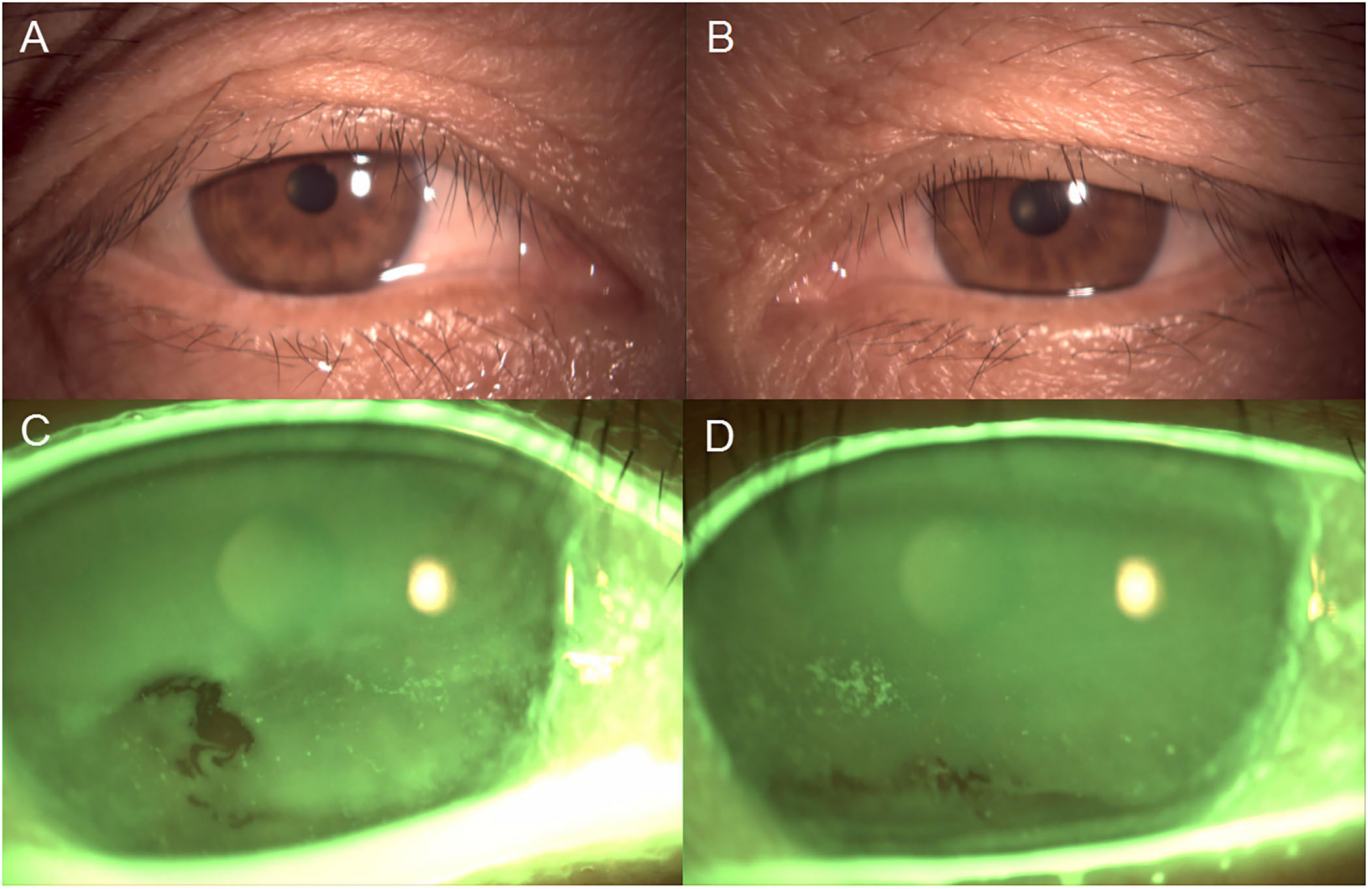
Slit-lamp photographs of both eyes after 1 week of treatment. **(A,B)** No lice, nits and fecal matter were found on the eyelashes and eyelids. **(C,D)** Corneal epithelial defects of both eyes had improved significantly.

One week later, no evidence of lice and/or nits on the eyelashes and scalp hair was found and the patient's symptoms had subsided completely. Meanwhile, corneal epithelial defects and BUT (up to 3 s) in each eye had also improved (Figure 2).

## Discussion

*Pthirus pubis* mainly infests the pubic hair (1). It is mainly transmitted in adults through sexual contact. However, in children, transmission occurs through close contact with infested contacts and/or fomites (2, 9). The infestation of the eyelashes and scalp is very rare (5–7). Until now, little has been known about the epidemiology of *Pthirus pubis* of the eyelashes and scalp. It is speculated that *Pthirus pubis* is transferred to the eyelashes and scalp by hands and/or fomites.

*Pthirus pubis* is stocky and discoid in shape (i.e., crab-like shape) with 2–3 mm in length, while two other subspecies of lice (i.e., head louse and body louse) are very similar, with slender shape and 3–4 mm in length (1, 2, 19). At present, in addition to dermoscopy and light microscope (4, 8), high-magnification slit-lamp microscopy is also helpful for the differential diagnosis (11, 15).

*Pthiriasis palpebrarum* is usually easily diagnosed by identification of crab-like lice and nits on the eyelids, but it may occasionally be misdiagnosed as blepharitis or conjunctivitis, especially in adults (12–15). On the other hand, although *Demodex* folliculorum blepharitis is a common parasitic disease (20), it is often overlooked in clinic. Therefore, patients presenting with pruritus and irritation symptoms of the eyelids and with resembling cylindrical sleeves on the eyelashes should be carefully examined (2).

Until now, several treatment options for *Pthiriasis palpebrarum* have been proposed, but none has been proven to be optimal (1–4). The simplest treatment is to directly trim or pluck of the eyelashes infected by crab lice and/or nits (1–4, 15). As for infestation of the scalp, in addition to cutting of the scalp hair, petrolatum jelly (Vaseline) and phenothrin rinse may be effective (6–8). In the present study, infected patient was advised to avoid close contact with other family members, and meanwhile to maintain good personal hygiene and environmental hygiene. In addition, Eto and his colleagues (7) described an outbreak of *Pthirus pubis* on the scalp, eyebrows and eyelashes of an elderly bedridden woman, who had been hospitalized for approximately 1 year. After a whole-body check, they found that 8 of 53 patients in the ward were infested. Therefore, close contacts should also be examined, especially her bedridden father.

**Figure 3:**
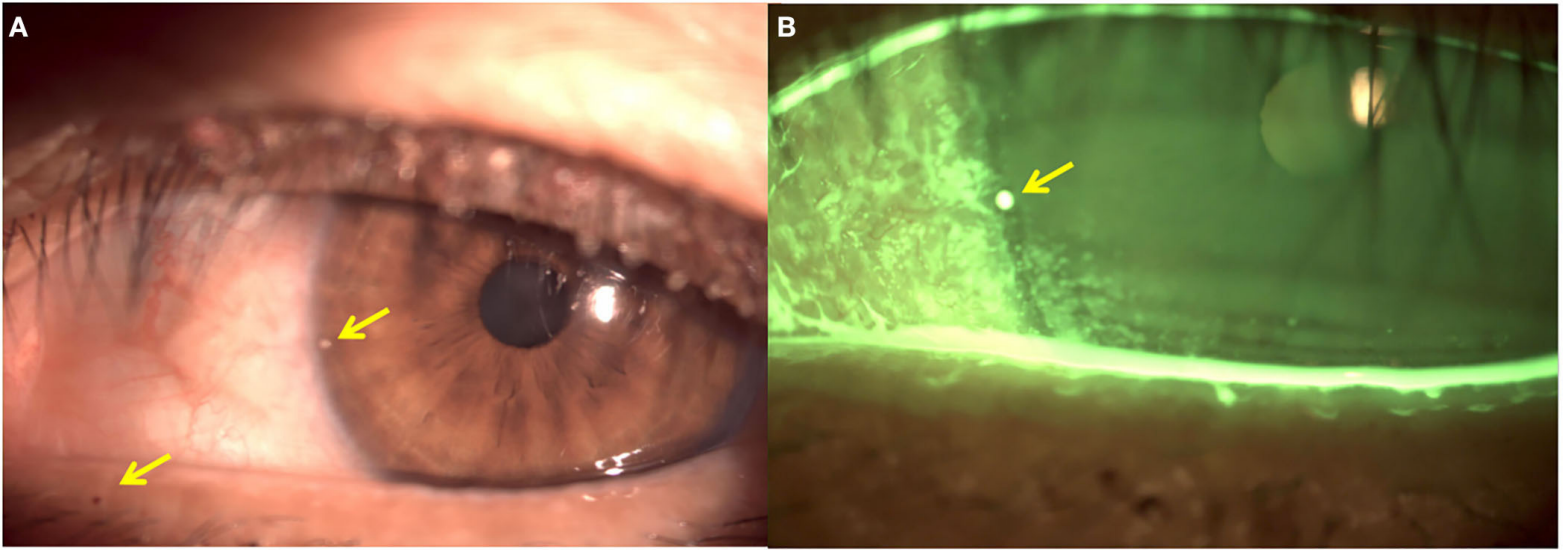
Slit-lamp photographs of left eye before treatment. **(A)** Fecal matter (yellow arrows) on the lid margin and the surface of cornea. **(B)** Corneal epithelial defects, and fecal matter (yellow arrow) on the surface of cornea.

In this case, given the patient's age, we speculate that the infestation of the eyelashes and scalp may have been transmitted during the care of her long-term bedridden father and then transferred to her granddaughter through close contact. In addition, corneal epithelial defects with short BUT may be directly caused by *Pthiriasis palpebrarum* (i.e., associated keratitis), or associated with dry eye. According to reports in the literature, Manjunatha et al. (17) reported a case of corneal epithelial keratitis associated with Pthiriasis palpebrarum. Ittyerah et al. (18) reported a 21-year old patient with marginal keratitis (sub-epithelial lesions) caused by *Pthiriasis palpebrarum*. They postulated that excretions and secretions of the lice could contribute to related keratitis. Also, we found that the fecal matter of louse tightly adhered to the surface of cornea before treatment (Figure 3). As for whether it is related to dry eye disease (DED), there have been few studies so far.

Since *Pthiriasis palpebrarum* is an infectious disease, in order to avoid cross-transmission, we didn't evaluate other tear parameters except BUT, such as Schirmer's test, tear meniscus height (TMH) and lipid layer thickness. Despite all this, according to the TFOS DEWS II Diagnostic Methodology report (21), this case might have dry eye disease (DED). But after treatment without artificial tears, the improvement of corneal involvement and the increase in BUT suggested that the findings were in favor of pthiriasis rather than DED. However, the BUT after treatment was still below normal. In summary, the patient might have concomitant DED. In order to further investigate the relationship between *Pthiriasis palpebrarum* and DED, a few weeks later, we called her for a follow-up, but unfortunately the patient refused to come to the hospital because of no ocular discomfort and in view of the coronavirus disease 2019 (COVID-19) pandemic. Therefore, further studies are warranted to provide more information.

Finally, it is important to know that the clinical manifestations of *Pthiriasis palpebrarum* may be similar to those caused by other diseases, such as blepharitis and blepharoconjunctivitis (10), but eyelashes with nits and/or lice are helpful for differential diagnosis.

## Conclusion

This rare case suggests that patients presenting with pruritus and irritation symptoms of the eyelids and with or without resembling cylindrical sleeves on the eyelashes should be carefully examined with a slit-lamp microscopy. Besides removal of the lice and nits, attention should be paid to the treatment of corneal epithelial injury associated with *Pthiriasis palpebrarum*.

## Data availability statement

The original contributions presented in the study are included in the article/Supplementary material, further inquiries can be directed to the corresponding author.

## Ethics statement

Written informed consent was obtained from the individual(s) for the publication of any potentially identifiable images or data included in this article.

## Author contributions

D-HW carried out the collection of the reported studies and the acquisition of data, with input from X-QL. D-HW conceived the study, made the interpretation of the information and drafted the initial manuscript, and reviewed and revised the manuscript. All authors contributed to the article and approved the submitted version.

## Funding

This work was supported by the Shanghai Municipal Commission of health and family planning (grant number: 20194Y0246), and the Young Talent Program of LongHua Hospital Shanghai University of Traditional Chinese Medicine (grant number: RC-2020-01-08).

## Conflict of interest

The authors declare that the research was conducted in the absence of any commercial or financial relationships that could be construed as a potential conflict of interest.

## Publisher's note

All claims expressed in this article are solely those of the authors and do not necessarily represent those of their affiliated organizations, or those of the publisher, the editors and the reviewers. Any product that may be evaluated in this article, or claim that may be made by its manufacturer, is not guaranteed or endorsed by the publisher.
